# Biofilm extracellular polysaccharides degradation during starvation and enamel demineralization

**DOI:** 10.1371/journal.pone.0181168

**Published:** 2017-07-17

**Authors:** Bárbara Emanoele Costa Oliveira, Jaime Aparecido Cury, Antônio Pedro Ricomini Filho

**Affiliations:** Department of Physiological Science, Piracicaba Dental School—University of Campinas, Piracicaba, São Paulo, Brazil; University of Florida, UNITED STATES

## Abstract

This study was conducted to evaluate if extracellular polysaccharides (EPS) are used by *Streptococcus mutans (Sm)* biofilm during night starvation, contributing to enamel demineralization increasing occurred during daily sugar exposure. *Sm* biofilms were formed during 5 days on bovine enamel slabs of known surface hardness (SH). The biofilms were exposed to sucrose 10% or glucose + fructose 10.5% (carbohydrates that differ on EPS formation), 8x/day but were maintained in starvation during the night. Biofilm samples were harvested during two moments, on the end of the 4^th^ day and in the morning of the 5^th^ day, conditions of sugar abundance and starvation, respectively. The slabs were also collected to evaluate the percentage of surface hardness loss (%SHL). The biofilms were analyzed for EPS soluble and insoluble and intracellular polysaccharides (IPS), viable bacteria (CFU), biofilm architecture and biomass. pH, calcium and acid concentration were determined in the culture medium. The data were analyzed by two-way ANOVA followed by Tukey’s test or Student's *t*-test. The effect of the factor carbohydrate treatment for polysaccharide analysis was significant (p < 0.05) but not the harvest moment (p > 0.05). Larger amounts of soluble and insoluble EPS and IPS were formed in the sucrose group when compared to glucose + fructose group (p < 0.05), but they were not metabolized during starvation time (S-EPS, p = 0.93; I-EPS, p = 0.11; and IPS = 0.96). Greater enamel %SHL was also found for the sucrose group (p < 0.05) but the demineralization did not increase during starvation (p = 0.09). In conclusion, the findings suggest that EPS metabolization by *S*. *mutans* during night starvation do not contribute to increase enamel demineralization occurred during the daily abundance of sugar.

## Introduction

Dental caries is a biofilm-sugar related disease that depends on biofilm accumulation on tooth surface and its frequent exposure to dietary carbohydrates [1]. The cariogenic biofilm forms and grows on dental surfaces in a dynamic condition in which the exposure to dietary carbohydrates occurs intermittently [2]. During the day, the biofilm is frequently exposed to short periods of great amount of carbohydrates, followed by long periods of non-exposure between the meals and overnight. These episodes are known as "feast" and "famine" periods and they are determinant for bacterial metabolism and biofilm growth [3, 4, 5]. In “feast” periods, bacterium such as *S*. *mutans* is able to store the excess of available carbohydrate as intracellular polysaccharides (IPS), which act as an energy reserve source in “famine” periods [6, 7]. Besides the IPS storage, glucosyl- and fructosyltransferases enzymes produced by *S*. *mutans* synthesize extracellular polysaccharides (EPS) when sucrose is the available carbohydrate [8, 9, 10].

EPS produced from sucrose contribute to microbial attachment and shifts on matrix tridimensional organization, mainly enhancing its porosity [11, 12, 13]. This structural change favors acid diffusion through the biofilm and pH fall next to the tooth surface, increasing enamel demineralization [14]. Additionally, it has been suggested that EPS could be an extracellular energy reserve, in which soluble glucans and fructans could be degraded by specific hydrolases, releasing glucose and fructose to be metabolized, producing acids [10, 15, 16, 17].

Once the degradation of EPS might occur during "famine" periods, the acid produced in a non-removed biofilm may extend the enamel demineralization especially during the night when salivary flow rate is low [18]. Furthermore, the effect of EPS metabolization on enamel demineralization has not been experimentally shown. The degradation of EPS has only been studied using planktonic cells [19, 20] and it was already reported that isolated or non-adsorbed enzymes could not simulate what happens in an organized biofilm [12, 21, 22]. Moreover, previous studies indicated that bacteria under starvation moments would have a different metabolic activity from those grown under constant nutrient exposure [23,24].

Therefore, considering that there is no data evaluating the effect of EPS degradation on enamel demineralization, the present study was carried out using a *S*. *mutans* cariogenic biofilm model subjected to intermittent periods of abundance and starvation of sucrose or equimolecular mixture of glucose and fructose. While sucrose is a source for IPS and EPS storage during energy abundance, glucose and fructose are only a source for IPS synthesis. Thus, it was possible to evaluate the degradation of EPS as an energy source during overnight starvation of carbohydrate and the consequent effect on enamel demineralization.

## Materials and methods

### Experimental design

An *in vitro*, randomized and blinded factorial (2x2) study was conducted using a validated cariogenic biofilm model [5, 25, 26]. The study was approved by the Research and Ethics Committee of the Piracicaba Dental School, University of Campinas (142/2014). The factors under study were type of carbohydrate (sucrose or a mixture of glucose + fructose) to feed the biofilms during the day, and biofilm harvest moment (at the end of the day after the last sugar treatment, situation of sugar abundance, or after overnight starvation), generating 4 experimental groups: (1) glucose + fructose abundance, (2) glucose + fructose starvation, (3) sucrose abundance, and (4) sucrose starvation. Sucrose and glucose + fructose are acidogenic carbohydrates and are stored as IPS, but only sucrose is substrate for EPS formation. *S*. *mutans* UA159 biofilms were formed on bovine enamel slabs, selected by surface hardness and randomized into the experimental groups (n = 12/group). The saliva-coated slabs were used to biofilms grown in ultrafiltered tryptone-yeast extract broth (UTYEB). The biofilms were exposed 8 times a day to 5.25% glucose + 5.25% fructose solution or 10% sucrose solution. Biofilm samples were harvested during two moments, at the end of the 4^th^ day and on the morning of the 5^th^ day, conditions of sugar abundance and starvation, respectively. Intracellular and extracellular polysaccharides (soluble and insoluble), biofilm dry weight and the number of viable cells were analyzed. During the biofilm growth, the culture medium was changed twice daily and the pH and calcium concentration were measured. The concentration of organic compounds (acids and ethanol) present in the medium were also evaluated. Enamel demineralization was calculated by percentage of surface hardness loss (%SHL). Confocal laser scanning microscopy was used to visualize the biofilm organization (bacterial cells and EPS), the images were three-dimensional reconstructed and the biomass (cells and EPS quantified together) was calculated. The hypothesis under study was that EPS are metabolized during night starvation, increasing enamel demineralization occurred during daily sucrose exposure. Three independent experiments were carried out and the data were statistically analyzed according to the factorial design of this study, considering enamel slab as a statistical block (n = 12).

### Enamel slabs preparation

Enamel slabs (4 x 7 x 1 mm) were obtained from bovine incisors [5]. The teeth were obtained from a local slaughterhouse, which is regulated by national legislation to follow sanitary standards. The bovine incisors were not collected for research purpose, and they were obtained after slaughtering. The teeth crowns were sectioned using a low-speed diamond blade to obtain enamel slabs. The surfaces were ground and polished using aluminum oxide abrasive papers (#400, #600 and #1200) and 1 μm diamond paste in a grinder machine (Phoenix Beta, Buehler, USA). The surface hardness (SH) was determined using a Knoop indenter (Future-Tech FM, Kawasaki, Japan) in which three indentations spaced 100 μm from each other were performed with 50 g load for 5 s on the polished surface center. The slabs with intra-variability higher than 10% were excluded and the selected slabs were randomized into the groups. The slabs were placed in 24-well culture plates, in vertical position using a metallic holder, and submitted to sterilization by exposure to ethylene oxide.

### Biofilm model

The cariogenic biofilm model was previously described by Ccahuana-Vasquez and Cury (2010). *Streptococcus mutans* UA159 reference strain [27] was used in the experiment and cultures stored at -80°C were first grown on Columbia blood agar (CBA). To prepare the inoculum, *S*. *mutans* colonies were transferred to ultrafiltered tryptone-yeast extract broth (UTYEB) supplemented with 1% glucose and incubated overnight at 37°C, 10% CO_2_. The cells were centrifuged, washed with saline solution, and resuspended in fresh UTYEB. The cell suspension was standardized in a spectrophotometer at OD_600_ of 1.6 ± 0.5 to obtain the bacterial inoculum. Prior to bacterial cell adhesion, the slabs were immersed in human saliva to form the acquired pellicle. Fresh stimulated whole saliva was collected from the same two healthy donors for each experiment. Salivary flow was stimulated by chewing a plastic paraffin film and the saliva was collected in 50 mL polypropylene tubes on ice for 30 minutes. The saliva was pooled, centrifuged (10,000 *g*, 4°C, 10 min) and filtered (0.22 μm) [25]. The enamel slabs were immersed into human saliva at 37°C for 30 min [5] to form the acquired pellicle. Saliva-coated slabs were transferred to wells containing 2 mL of fresh UTYEB with 1% glucose and the bacterial inoculum (1:500 v/v), and were incubated for 8 h to promote initial adhesion of the microorganisms. Only in the adhesion phase, the UTYEB pH 7.0 was strongly buffered (10x higher than the usual phosphate concentration) to avoid pH drop and enamel demineralization during this phase. After adhesion, the slabs were transferred to fresh UTYEB pH 7.0 with 0.1 mM glucose (salivary basal concentration) and incubated overnight at 37°C, 10% CO_2_ for 16 h. At the beginning of 2^nd^ day, the biofilms were exposed to episodes of “feast” and “famine” comprised of 8 daily exposures to carbohydrate solutions: 10% sucrose or a combination of 5.25% glucose and 5.25% fructose at predetermined times (08:00, 09:30, 11:00, 12:00, 13:30, 15:00, 16:00 and 17:30 h) for 3 min [25, 26]. After the cariogenic challenge, enamel slabs were rinsed 3 times in 0.9% NaCl solution, and placed back into the culture media. The culture media was changed twice daily, before the first challenge and after the last one. The pH was immediately evaluated after the change and the medium of each well was stored individually in microcentrifuge tubes at -80°C for later quantification of organic compounds and calcium concentration. At the end of the 4^th^ day of biofilm formation, just after the last treatment, the biofilms were rinsed and remained during 10 min in fresh saline solution as standardized in a pilot study. This step was to avoid carrying any carbohydrate of the medium that could interfere in polysaccharide analysis. Half of the slabs with biofilms were collect for abundance condition evaluation. The other half was placed in fresh culture medium without glucose basal concentration, and at beginning of the 5^th^ day, the biofilms were collected in starvation condition, after 16 h of night fasting (overnight starvation). The biofilm harvest was performed by sonication at 7 watts for 30 s as described by Ccahuana-Vasquez and Cury (2010). The slabs were used to evaluate enamel demineralization and the suspension was used for biofilm analyses.

### Biofilm analyses

An aliquot of 100 μL of the biofilm suspension was ten-fold serially diluted in saline solution until 1:10^7^. Two drops of 20 μL of each dilution were plated on Todd-Hewitt broth (THB) plus agar and incubated at 37°C, 10% CO_2_ for 48 h and the counts of the colony forming unit (CFU) was performed with the aid of a stereoscopic microscope [5, 28, 29]. The extraction of extracellular polysaccharides, soluble (S-EPS) and insoluble (I-EPS), and intracellular polysaccharides (IPS) was performed as described by Aires et al. (2008) from an aliquot of 400 μL of the sonicated biofilm suspension [30]. The amount of total carbohydrates was quantified by the phenol sulfuric method [31] using glucose as standard. Another aliquot of 400 μL of the sonicated biofilm suspension was added in pre-weigh microcentrifuge tubes to perform the biofilm dry weight analysis. The dry weight was determined by the difference between the final and initial weight of the microcentrifuge tubes [5, 29].

### Culture medium analysis

The pH of the culture medium was evaluated twice a day at each medium change as an indicator of biofilm acidogenicity. The pH measurement was performed using a pH microelectrode (Accumet; Cole-Parmer, USA) coupled to a pHmeter (Procyon SA-720, Olímpia, Brazil) calibrated with pH standards of 4.0 and 7.0, performed directly inside the wells, just after the medium change. Then, the culture medium was stored for calcium concentration analysis and organic compounds quantification. The Arsenazo III colorimetric method [32] was used to assess calcium concentration in 10 μL of medium from each sample. The absorbance was read at a wavelength of 650 nm in 96-well plates using a Multiskan Spectrum (Thermo Scientific) microplate reader [25]. The results were expressed as calcium concentration (mM) in function of the time of biofilm formation, and as cumulative calcium (μg) released from enamel (the sum of total quantity of calcium released from enamel to the medium until the harvest moments of abundance and starvation). The concentration of organic compounds (mM) was also performed at the two harvest moments. High Performance Liquid Chromatography (Alliance 2795, Waters, USA) was used with a refractive index detector and an Aminex HP-87H column (Bio-Rad Laboratories, USA) [33]. Standard curves of lactic, acetic and formic acids, and ethanol were used to calculate the concentration of each organic compound in samples. Triplicates of two independent experiments were used (n = 6).

### Enamel demineralization assessment

After biofilm removal, the slabs were used to evaluate the enamel demineralization. The SH was performed again by three indentations 100 μm apart from the initial SH measurement and the percentage of surface hardness loss (% SHL) was calculated as follows: (baseline SH—SH after assay × 100)/baseline SH [14].

### Confocal laser scanning microscopy (CLSM)

To visualize the architecture of biofilms formed under exposure to glucose + fructose or sucrose at abundance and starvation moments, additional enamel slabs were used. The extracellular matrix was labeled during the biofilm development with 1 μM Alexa Fluor 647—dextran conjugate (molecular weight, 10,000; Excitation 650 nm/ Emission 668 nm; Thermo Scientific, USA) added to the culture medium. At the end of development, the biofilms were fixed in 4% paraformaldehyde (PBS, pH 7.2) for 30 min. *S*. *mutans* cells were stained with 2.5 μM SYTO-9 green fluorescent nucleic acid (Excitation 485 nm/ Emission 498 nm; Thermo Scientific, USA) under protection from light for 20 min [34, 35]. The stained biofilms were mounted on coverslips containing 10 μL of Mowiol, a glycerol based mounting medium for fluorescent staining [36]. The stained biofilms were examined using a DMI 6000 CS inverted microscope coupled to TCS SP5 computer-operated confocal laser scanning system (Leica Microsystems CMS, Mannheim, Germany) using 40x oil immersion objective (numeric aperture 1.25). An Ar-ion laser tuned at 488 nm and a He-Ne laser at 633 nm were used for excitation. Two enamel slabs were used for each condition evaluated. Five randomly sites were selected in the central area of the biofilms, avoiding the edges. Series of images were obtained from the enamel surface to the biofilm top at 1 μm distance each in the Z axis [37]. The images of the biofilms were used for three-dimensional reconstruction with the ImageJ software [38], and to calculate the biofilm biomass (μm^3^/μm^2^) using the Comstat 2 software [39, 40]. Although the images of bacteria and EPS were obtained from different channels in CLSM, the biomass was calculated considering cells and EPS as a single channel. Therefore, biomass represents bacterial cells and EPS together.

### Statistical analysis

Data were analyzed by two-way ANOVA, considering the factors carbohydrate (glucose + fructose or sucrose) and biofilm harvest moment (abundance or starvation), followed by Tukey’s HSD Test. Student's *t*-test was used to evaluate differences between the carbohydrates for pH and calcium concentration values at each time point (8, 24, 32, 48, 56, 72, 80 and 96 h). The statistical analysis was done using SAS software (SAS Institute Inc., version 8.01, Cary, N.C., USA) employing a significance level fixed at 5%. Assumptions of homogeneity of variances and normal distribution of errors were checked for all tested response variables using the Kolmogorov-Smirnov test. Data that violated the assumptions were transformed to square-root (biofilm dry weight, %SHL and cumulative calcium released from enamel) and to log_10_ (CFU counts, CFU counts/mg biofilm dry weight, polysaccharides, organic compounds).

## Results

**Fig 1:**
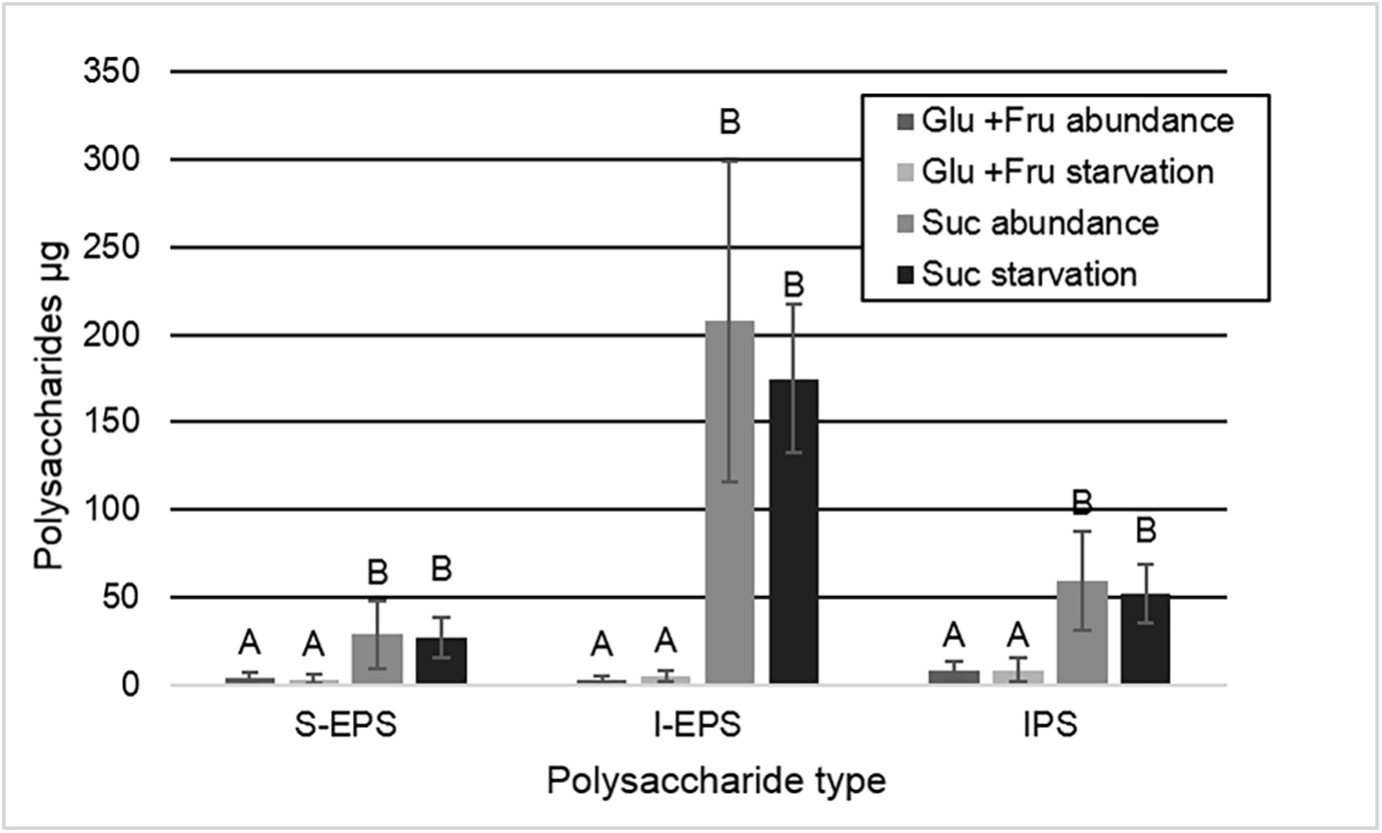
Amounts (μg) per enamel slab of extracellular polysaccharides, soluble (S-EPS) and insoluble (I-EPS), and intracellular polysaccharides (IPS) according to the treatments (glucose + fructose or sucrose) and the harvest moment (abundance or starvation). Distinct capital letters indicate significant statistically differences among groups for each polysaccharide type (p < 0.05) (Mean ± SD; n = 12).

For the amounts of polysaccharides, the statistical analyses showed a significant difference for the factor carbohydrate (p < 0.05), however no differences were observed for the harvest moment (S-EPS, p = 0.93; I-EPS, p = 0.11; and IPS = 0.96) and for the interaction effect between the two factors (carbohydrate and harvest moment) (S-EPS, p = 0.82; I-EPS, p = 0.08; and IPS = 0.70). The same statistical pattern was observed for the variables: biofilm dry weight, CFU per biofilm dry weight, %SHL and biomass. The amounts of intracellular (IPS), and soluble (S-EPS) and insoluble (I-EPS) extracellular polysaccharides in biofilms formed under daily exposure to sucrose were higher, when compared to those found in biofilms exposed to glucose + fructose (Fig 1) (p < 0.05).

**Fig 2:**
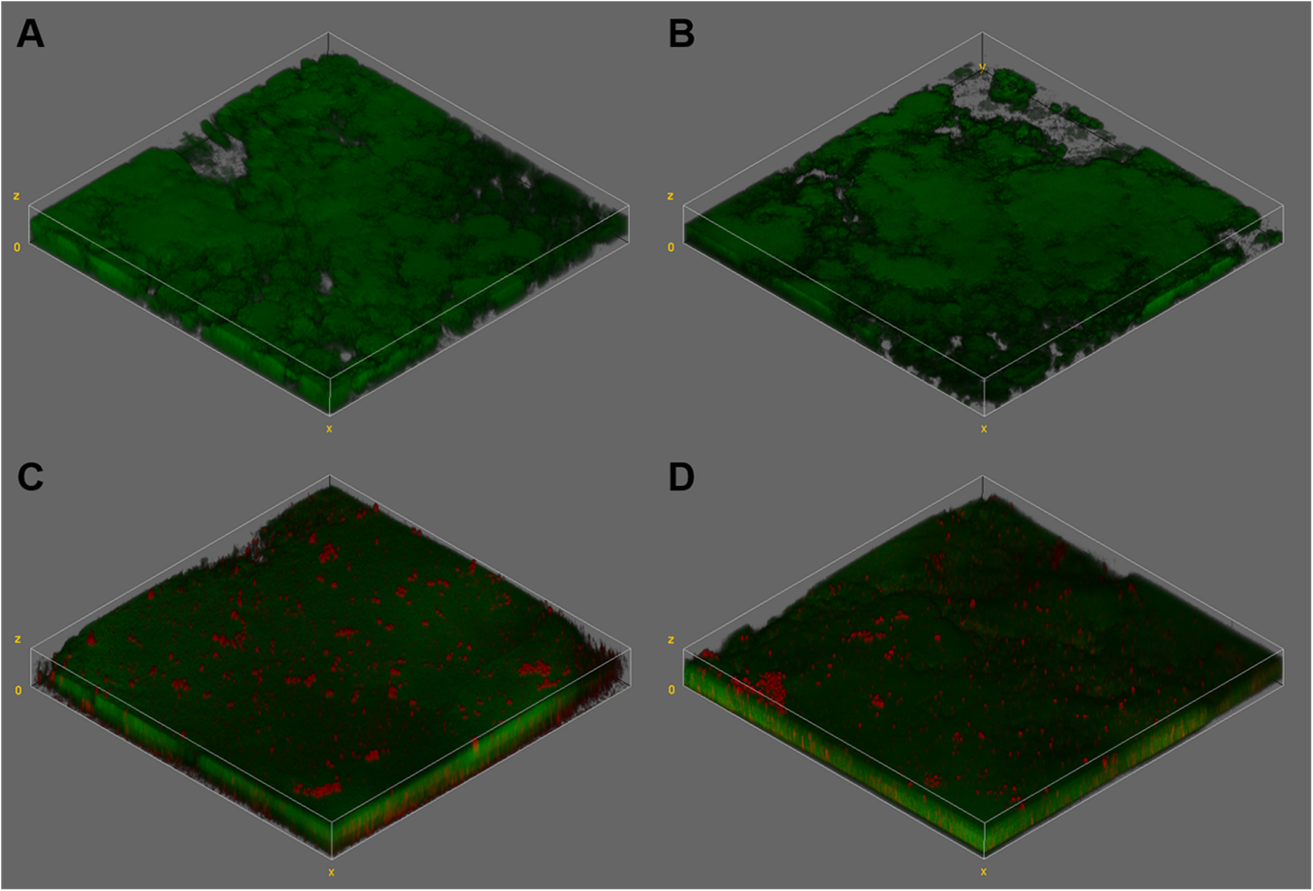
**Tridimensional reconstruction of CLSM images of *S*. *mutans* biofilms formed under exposure to glucose + fructose (A and B) or sucrose (C and D) 8x/daily**. Images A and C show biofilms visualized at abundance moment, while images B and D at starvation moment. In green, *S*. *mutans* cells stained with SYTO 9. In red, extracellular polysaccharides labeled with Alexa Fluor 647—dextran conjugate. Oil immersion objective of 40x (numeric aperture 1.25).

The biofilm dry weight was much higher when the biofilm was formed under sucrose exposure than those formed with glucose + fructose (p < 0.05), and no significant change was observed after overnight starvation. On the other hand, there was no effect for the carbohydrate (p = 0.20) and neither for the harvest moment (p = 0.07) on the CFU counts (Table 1). When CFU counts were normalized by the biofilm dry weight, a greater number of viable cells per volume of biofilm was observed in the group exposed to glucose and fructose (p < 0.05) (Table 1). CLSM images showed extracellular polysaccharides (red) only synthesized in biofilms exposed to sucrose (Fig 2). In glucose + fructose group, only *S*. *mutans* cells (green) were visualized. It was also possible to observe that biofilm under sucrose treatment presented higher biomass (Table 1 and Fig 2), which is in accordance to EPS analysis (Fig 1) and biofilm dry weight (Table 1).

**Table 1:** Mean ± SD (n = 12) of biofilm dry weight (mg), CFU counts (Log_10_), CFU counts /mg biofilm dry weight (CFU Log_10_/mg), %SHL, cumulative calcium released from enamel (μg) and biomass (μm^3^/μm^2^) according to the treatments (glucose + fructose or sucrose) and the harvest moment (abundance or starvation).

		Analysis					
Carbohydrate	Biofilm harvest moment	Biofilm dry weight (mg)^¤^	CFU counts (CFU Log_10_) *	(CFU Log_10_)/mg biofilm dry weight *	%SHL^¤^	Cumulative Ca^++^ released from enamel (µg) ^¤^	Biomass ^$^ (μm^3^/μm^2^)
Glucose + Fructose	abundance	0.4 ± 0.2 ^A^	7.4 ± 1.1 ^A^	12.3 ± 1.1 ^A^	14.5 ± 7.1 ^A^	24.7 ± 14.8 ^A^	22.3 ± 5.9 ^A^
	starvation	0.6 ± 0.4 ^A^	7.8 ± 1.2 ^A^	15.4 ± 7.5 ^A^	11.9 ± 4.6 ^A^	17.1 ± 10.7 ^A^	23.7 ± 7.9 ^A^
Sucrose	abundance	1.6 ± 0.3 ^B^	7.6 ± 1.0 ^A^	4.8 ± .3.0 ^B^	41.4 ± 6.5 ^B^	85.1 ± 29.9 ^B^	36.2 ± 8.4 ^B^
	starvation	1.6 ± 0.3 ^B^	8.3 ± 0.5 ^A^	5.4 ± 1.2 ^B^	34.7 ± 7.7 ^B^	33.2 ± 24.4 ^A^	36.1 ± 7.1 ^B^

Distinct capital letters indicate significant statistically differences (p < 0.05) among groups for each variable (values within columns).

* The values were transformed to log_10._

^¤^ The values were transformed to square-root.

^$^ Biomass refers to bacterial cells and EPS

**Fig 3:**
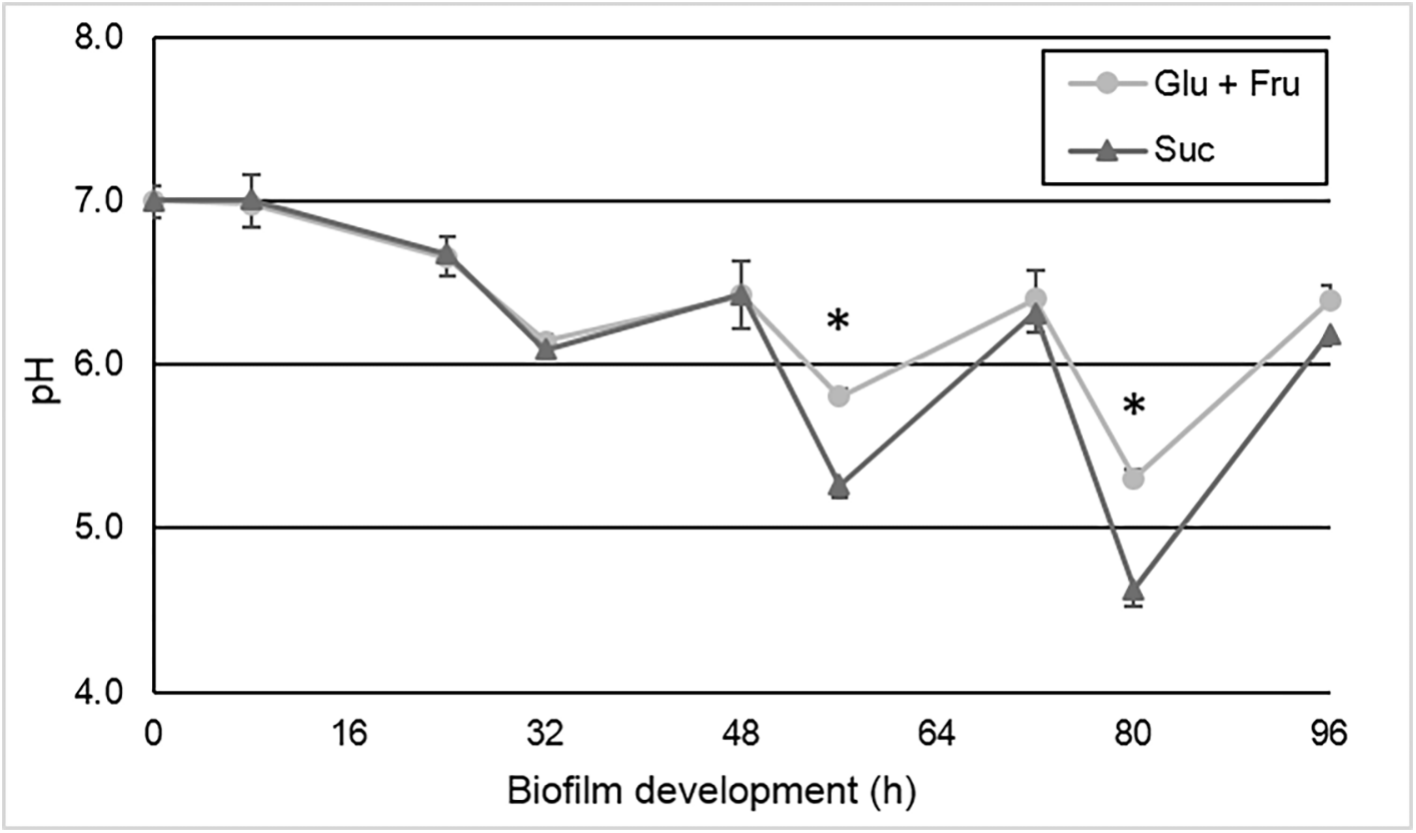
pH values of the culture medium according to the biofilm treatments (glucose + fructose or sucrose) and biofilm development time (h) as an indicator of biofilm acidogenicity. Time points at 80 h and 96 h refer to the harvest moment at abundance and starvation of carbohydrates, respectively. Asterisks indicate statistically significant difference between treatments at the time point evaluated (p < 0.05). (Means ± SD; n = 12).

**Fig 4:**
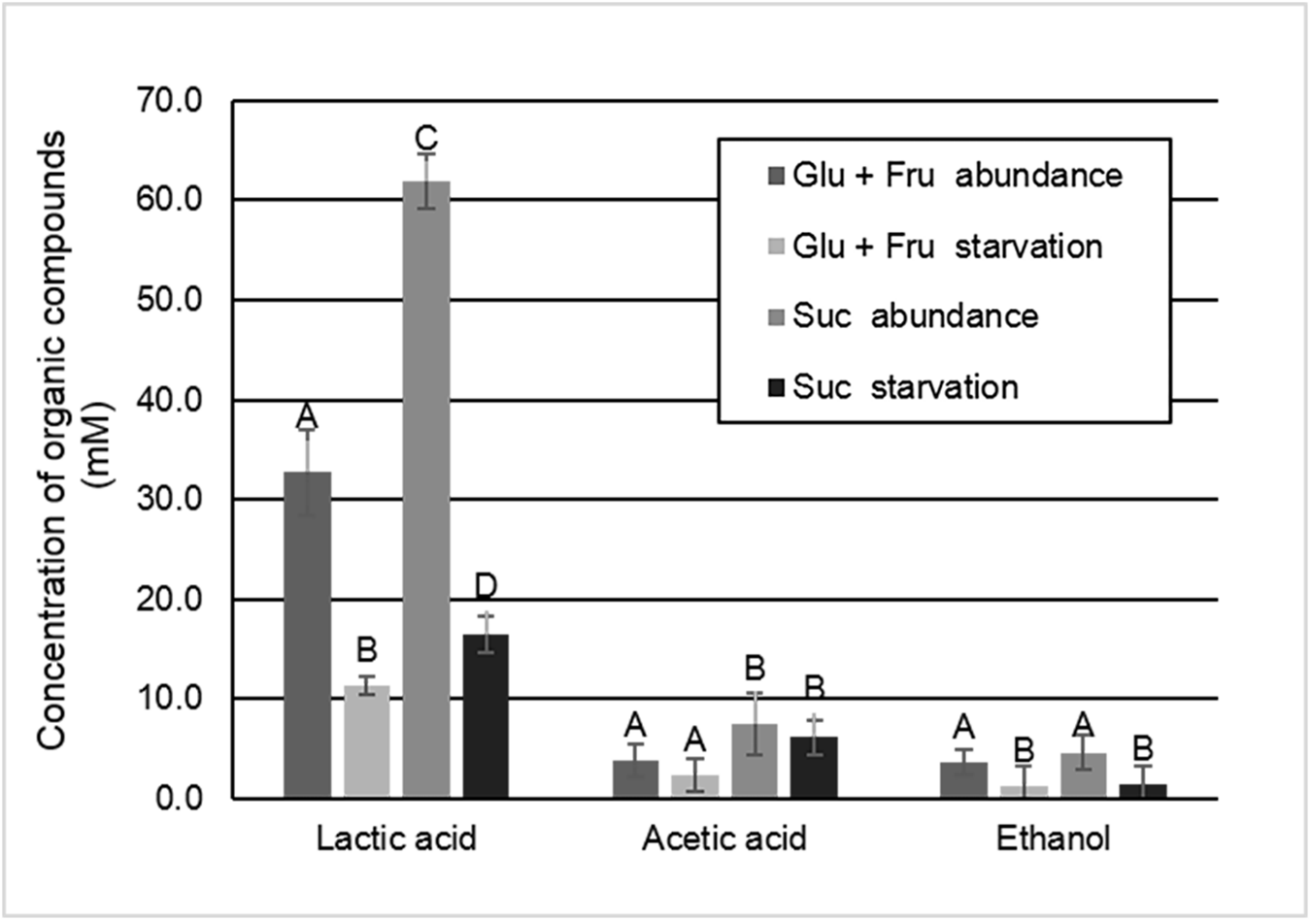
Concentration of organic compounds (mM) in the culture medium according to the biofilm treatments (glucose + fructose or sucrose) and the harvest moment (abundance or starvation). Distinct capital letters indicate significant statistically differences among groups for each type organic compound (p < 0.05). (Mean ± SD; n = 6).

The pH values of the culture medium were lower in the group exposed to sucrose. This difference was observed from 56 h, which corresponds to 2^nd^ day of biofilm development under cariogenic challenge, when it started to be mature [36] (Fig 3). Lower amount of acids was produced by *S*. *mutans* when biofilm was formed under glucose + fructose exposure, when compared to sucrose (p < 0.05). Among the acids, lactic acid was the most produced in both treatment groups, showing significant effect for the factors carbohydrate and harvest moment (p < 0.05). The values of lactic acid produced by biofilms exposed to sucrose were 2 times higher than those observed in the group treated with the combined monosaccharides (p < 0.05). Acetic acid was produced in small amounts and only the carbohydrate factor had a significant effect (p < 0.05). Ethanol was also produced as a final compound of the carbohydrate metabolism being significant only to the harvest moment factor (p < 0.05) (Fig 4). In starvation condition, acid production was lower for both glucose + fructose and sucrose groups, but it occurred even after removal of all carbohydrate sources (p < 0.05).

**Fig 5:**
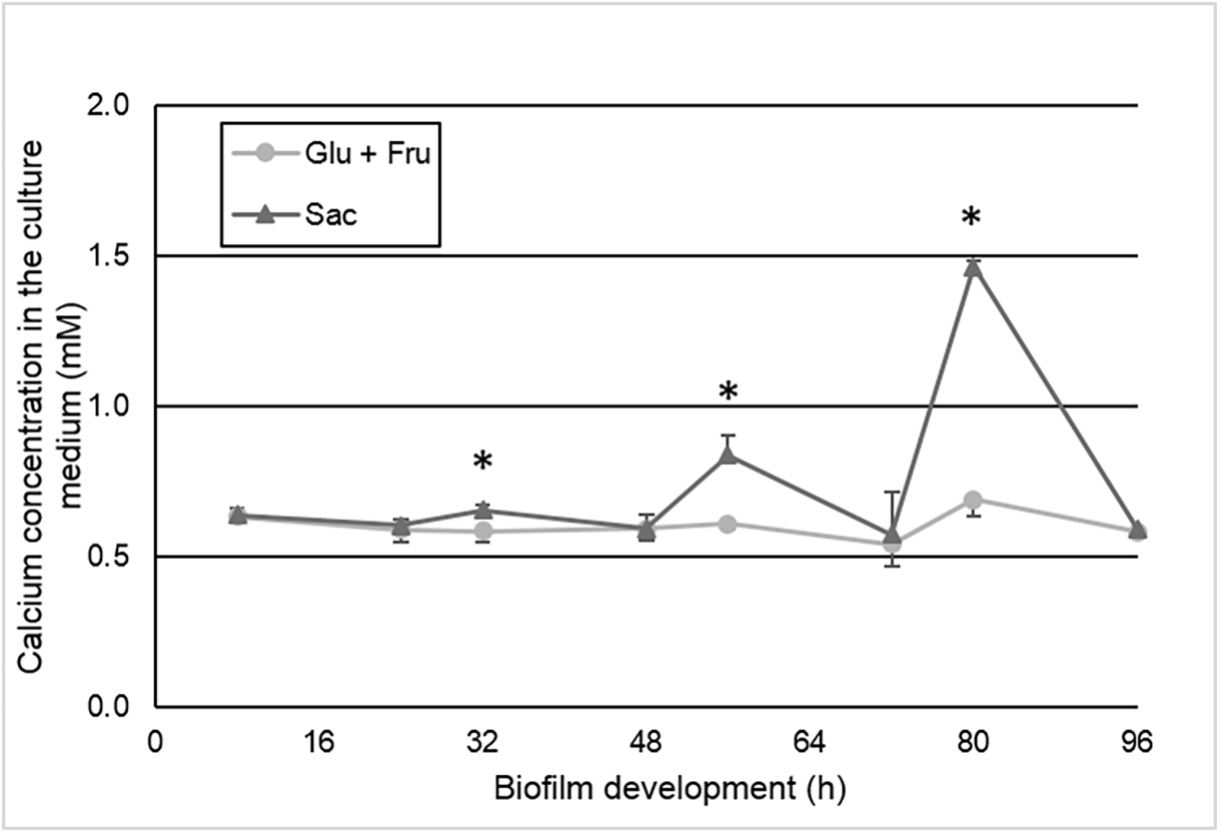
Calcium concentration (mM) in the culture medium according to the biofilm treatments (glucose + fructose or sucrose) and biofilm development time (h). Time points at 80 h and 96 h refer to the harvest moment at abundance and starvation of carbohydrates, respectively. Asterisks indicate statistically significant difference between treatments at the time point evaluated (p < 0.05). (Mean ± SD, n = 12).

The calcium concentration in culture medium during biofilm development was higher in sucrose group (p < 0.05). This higher quantity released from enamel was seen from 32 h of biofilm development and became more evident with the biofilm maturation (Fig 5), mainly at the end of the 4^th^ day (abundance condition) (p < 0.05). This behavior was consistent with the pH (Fig 3) and %SHL (Table 1) data. Higher %SHL was observed in enamel slabs in the sucrose group, which differed statistically from the group treated with the combined monosaccharides (p <0.05). Under sucrose exposure, the enamel slabs lost around 30 to 40% of hardness, while those exposed to glucose + fructose lost around 15%. No significant effect was observed in relation to harvest moment to the %SHL data (Table 1).

The cumulative calcium released from enamel showed higher amount of calcium released when the biofilm was exposed to sucrose, however only in the harvest moment of abundance (p < 0.05) (Table 1). The cumulative calcium values from the group exposed to sucrose at starvation period were similar to the groups exposed to glucose + fructose, regardless of the harvest moment (abundance or starvation).

## Discussion

In this study, we used a validated *S*. *mutans* biofilm model for caries study [5] to evaluate the degradation of extracellular polysaccharides (EPS) as an energy source during overnight starvation of carbohydrate and the consequent effect on enamel demineralization. Once this approach has not been performed using biofilms, this study seems to be a pioneer in the field. The experimental design using different carbohydrates allowed us to develop biofilms with EPS (sucrose) or without them (glucose + fructose), however, for both groups, bacterial cells could also storage intracellular polysaccharides (IPS), which could also be used as an energy source. Our findings showed no reduction in the amounts of EPS and IPS (Fig 1), and no increase in enamel demineralization after the overnight period of starvation (Table 1). The non-reduction of EPS and IPS quantities may indicate that they were not metabolized during the starvation period, or that they were metabolized in a small amount, just to maintain the bacterial basal metabolism, causing no additional damage to the enamel.

The lack of increase in enamel demineralization was confirmed not only by the percentage of surface hardness loss (%SHL) (Table 1), but also by the calcium concentration in the culture medium (Fig 5). Thus, the known cariogenicity of sucrose [8, 10, 41, 42] was evidenced in our study. The sucrose group presented %SHL almost 3 times higher than the glucose + fructose group (p < 0.05) (Table 1). However, no difference in the %SHL was observed between the abundance and starvation moments for each group. In order to verify the enamel demineralization using a more sensitive analysis, we also evaluated the calcium concentration in the culture medium. Although higher amounts of calcium in the medium were observed for the sucrose group (Fig 5; p < 0.05) after the abundance period (80 h), no increase (p > 0.05) in the calcium concentration was detected when both groups were compared after the overnight starvation period (96 h). This finding could strengthen and confirm that there was no increase in enamel demineralization during the starvation period by the EPS or IPS use.

Calcium kinetics (Fig 5) inversely follows the pH’s dynamic (Fig 3) occurred into biofilm [25]. Therefore, higher acid production caused higher enamel demineralization, releasing more calcium from the enamel to the culture medium. Lower pH values were observed for the group exposed to sucrose after the abundance period from the second day of cariogenic challenge (56 and 80 h) (Fig 3). Even using solutions with equimolar concentration of carbohydrates, the pH values for the sucrose group were lower than the glucose + fructose group. The lower pH is explained by the EPS in the matrix that are only synthesized in the presence of sucrose [8]. It is known that EPS change the biofilm structure by the increase of the matrix porosity, which favors acid diffusion and pH drops nearby the enamel surface [4, 11]. In addition, micro-compartments formed within the biofilm matrix could generate heterogeneous environments keeping the pH low for longer periods [13, 43], increasing the cariogenic challenge. In our study, during the cariogenic challenges, the acids produced by bacteria in the biofilm caused the enamel demineralization [44]. Calcium was continuously released from enamel to the biofilm, and from the biofilm to the medium. The same occurred for the acids produced. Therefore, the calcium and acid concentration in the medium reflect the cariogenic process occurred on the enamel surface in contact with the biofilm.

The higher acid production for the biofilm exposed to sucrose (Fig 4) cannot be explained either by the counts of *S*. *mutans* cells, because they were similar for the sucrose and glucose + fructose groups at both harvest moment (Table 1). The difference in the sucrose group was the presence of EPS in the matrix. In addition to modifying the biofilm architecture [43], the synthesized EPS also increased the biofilm volume (Table 1). The biofilm formed under sucrose exposure was 3 times heavier than the glucose + fructose group (p < 0.05) (Table 1), which could represent from 10 to 40% of the biofilm dry weight [10, 12]. Tridimensional images of the biofilms (Fig 2) and biomass data (Table 1) also showed that difference (p < 0.05). Taking into account CFU counts normalized by biofilm dry weight and biomass data, it was possible to infer that more cells per volume were present in the biofilm exposed to glucose + fructose, because in this condition there were no EPS. On the other hand, the biofilm exposed to sucrose showed less cells per volume, suggesting EPS could determine the bacterial density in biofilms [17]. The cells dispersed into EPS matrix would be easily exposed to sugars, due to higher porosity of the biofilm [11], which could allow more acid production and IPS storage, when compared to the packed cells in the biofilm without EPS (glucose + fructose). This could also explain the great amount of IPS formed in the group exposed to sucrose (Table 1). Thus, the increase in biofilm cariogenicity can be better understood by changes occurred in matrix composition and structure, and not simply by the counts of bacterial cells in biofilms [43].

The presence of EPS in dental biofilm matrix has been reported as an important virulence factor for caries development [8, 13, 14, 41, 43]. A study reported increased expression of a gene related to EPS degradation (*dexA*) in *S*. *mutans* mature biofilms [42], and another showed the non-utilization of IPS when sucrose was present [6], both suggesting that EPS from biofilm matrix could be used as an extracellular energy source [15, 17]. It was already demonstrated in planktonic cultures that other oral microorganisms could produce EPS and degrade them [20]. However, the EPS produced by *Streptococcus mutans* seem to be far less soluble and more resistant to dextranase action [20]. EPS degradation was not observed in our study using a *S*. *mutans* biofilm model. One of the reasons why there was no difference for EPS quantities between the two harvest moments might be due to the starvation period. Maybe a longer fasting period could trigger the metabolization of polysaccharides by the cells to obtain energy. Another reason could be that bacterial cells just break and use small ending fragments of the EPS, which had not been detected by the phenol sulfuric method. Therefore, future studies using a longer period of starvation or a methodology using radiolabelled sucrose in the fructosyl (^3^H) and glucosyl (^14^C) moieties, instead of the phenol sulfuric method, might be useful to clarify EPS metabolization.

Considering that the used *S*. *mutans* biofilm model simulates the "feast” and “famine" episodes that occur into the mouth, the EPS metabolism during starvation may not be sufficient to increase the biofilm cariogenicity, but the high carbohydrate exposure frequency occurred during the day. In summary, the findings suggest that EPS metabolization by *S*. *mutans* during night starvation do not contribute to increase the enamel demineralization occurred during the daily abundance of sugar.

## References

[1] Fejerskov O. Changing paradigms in concepts on dental caries: consequences for oral health care. Caries Res 2004;38:182–191. doi: 10.1159/000077753 15153687 10.1159/000077753

[2] Marsh PD. Are dental diseases examples of ecological catastrophes? Microbiology 2003;149:279–294. doi: 10.1099/mic.0.26082-0 12624191 10.1099/mic.0.26082-0

[3] Carlsson J. Bacterial metabolism in dental biofilms. Adv Dent Res 1997;11:75–80. doi: 10.1177/08959374970110012001 9524445 10.1177/08959374970110012001

[4] Bowden GH, Hamilton IR. Survival of oral bacteria. Crit Rev Oral Biol Med 1998;9:54–85. 9488248 10.1177/10454411980090010401

[5] Ccahuana-Vásquez RA, Cury JA. *S*. *mutans* biofilm model to evaluate antimicrobial substances and enamel demineralization. Braz Oral Res 2010;24:135–41. 20658029 10.1590/s1806-83242010000200002

[6] Busuioc M, Mackiewicz K, Buttaro BA, Piggot PJ. Role of intracellular polysaccharide in persistence of *Streptococcus mutans*. J Bacteriol 2009;191:7315–22. doi: 10.1128/JB.00425-09 19801415 10.1128/JB.00425-09 PMC2786568

[7] Takahashi N. Oral Microbiome Metabolism: From "Who Are They?" to "What Are They Doing?" J Dent Res 2015;94:1628–1637. doi: 10.1177/0022034515606045 26377570 10.1177/0022034515606045

[8] Rölla G. Why is sucrose so cariogenic? The role of glucosyltransferases and polysaccharides. Scand J Dent Res. 1989;97:115–119. 2523085 10.1111/j.1600-0722.1989.tb01439.x

[9] Bowen WH. Do we need to be concerned about dental caries in the coming millennium? Crit Rev Oral Biol Med 2002;13:126–31. 12097355 10.1177/154411130201300203

[10] Paes Leme AF, Koo H, Bellato CM, Bedi G, Cury JA. The role of sucrose in cariogenic dental biofilm formation-new insight. J Dent Res 2006;85:878–887. doi: 10.1177/154405910608501002 16998125 10.1177/154405910608501002 PMC2257872

[11] Dibdin GH, Shellis RP. Physical and biochemical studies of *Streptococcus mutans* sediments suggest new factors linking the cariogenicity of plaque with its extracellular polysaccharide content. J Dent Res 1988;67:890–895. doi: 10.1177/00220345880670060101 3170900 10.1177/00220345880670060101

[12] Bowen WH, Koo H. Biology of *Streptococcus mutans*—derived glucosyltransferases: role in extracellular matrix formation of cariogenic biofilms. Caries Res. 2011;45:69–86. 10.1159/000324598 PMC3068567 21346355

[13] Koo H, Falsetta ML, Klein MI. The exopolysaccharide matrix: a virulence determinant of cariogenic biofilm. J Dent Res 2013;92:1065–1073. doi: 10.1177/0022034513504218 24045647 10.1177/0022034513504218 PMC3834652

[14] Cury JA, Rebelo MA, Del Bel Cury AA, Derbyshire MT, Tabchoury CP. Biochemical composition and cariogenicity of dental plaque formed in the presence of sucrose or glucose and fructose. Caries Res 2000;34:491–497. 11093024 10.1159/000016629

[15] Wood JM. The amount, distribution and metabolism of soluble polysaccharides in human dental plaque. Arch Oral Biol. 1967;12:849–858. 5231456 10.1016/0003-9969(67)90107-0

[16] Whiting GC, Sutcliffe IC, Russell RR. Metabolism of polysaccharides by the *Streptococcus mutans dexB* gene product. J Gen Microbiol 1993;139:2019–2026. doi: 10.1099/00221287-139-9-2019 7504068 10.1099/00221287-139-9-2019

[17] Colby SM, Russell RR. Sugar metabolism by mutans streptococci. Soc Appl Bacteriol Symp Ser 1997;26:80–88. 9436320

[18] Dawes C. Circadian rhythms in human salivary flow rate and composition. The J Physiol 1972; 220(3): 529–545 5016036 10.1113/jphysiol.1972.sp009721 PMC1331668

[19] Walker GJ, Hare MD, Morrey-Jones JG. Activity of fructanase in batch cultures of oral streptococci. Carbohydr Res 1983;113:101–112. 6839310 10.1016/0008-6215(83)88222-6

[20] Schachtele CF, Loken AE, Schmitt MK. Use of specifically labeled sucrose for comparison of extracellular glucan and fructan metabolism by oral streptococci. Infect Immun 1972;5:263–266. 4564402 10.1128/iai.5.2.263-266.1972 PMC422359

[21] Kopec LK, Vacca-Smith AM, Bowen WH. Structural aspects of glucans formed in solution and on the surface of hydroxyapatite. Glycobiology 1997;7:929–934. 9363435 10.1093/glycob/7.7.929

[22] Aires CP, Tenuta LMA, Carbonero ER, Sassaki GL, Iacomini M, Cury JA. Structural characterization of exopolysaccharides from biofilm of a cariogenic streptococci. Carbohydr Polym 2011;84:1215–1220.

[23] Huis in ‘t Veld JH, Backer Dirks O. Intracellular polysaccharide metabolism in *Streptococcus mutans*. Caries Res 1978;12:243–249 279404 10.1159/000260340

[24] Zhu M, Takenaka S, Sato M, Hoshino E. Influence of starvation and biofilm formation on acid resistance of *Streptococcus mutans*. Oral Microbiol Immunol 2001;16:24–27. 11169135 10.1034/j.1399-302x.2001.160104.x

[25] Fernández CE, Tenuta LM, Cury JA. Validation of a cariogenic biofilm model to evaluate the effect of fluoride on enamel and root dentine demineralization. PLoS one. 2016 1 5;11(1):e0146478 doi: 10.1371/journal.pone.0146478 26731743 10.1371/journal.pone.0146478 PMC4712139

[26] Fernández CE, Giacaman RA, Tenuta LM, Cury JA. Effect of the probiotic *Lactobacillus rhamnosus* LB21 on the cariogenicity of *Streptococcus mutans* UA159 in a dual-species biofilm model. Caries Res 2015;49:583–590. doi: 10.1159/000439315 26451810 10.1159/000439315

[27] Ajdić D, McShan WM, McLaughlin RE, Savić G, Chang J, Carson MB, et al Genome sequence of *Streptococcus mutans* UA159, a cariogenic dental pathogen. Proc Natl Acad Sci USA 2002;99:14434–9. doi: 10.1073/pnas.172501299 12397186 10.1073/pnas.172501299 PMC137901

[28] Tenuta LM, Ricomini Filho AP, Del Bel Cury AA, Cury JA. Effect of sucrose on the selection of mutans streptococci and lactobacilli in dental biofilm formed *in situ*. Caries Res 2006;40:546–549. doi: 10.1159/000095656 17063028 10.1159/000095656

[29] Koo H, Hayacibara MF, Schobel BD, Cury JA, Rosalen PL, Park YK, et al Inhibition of *Streptococcus mutans* biofilm accumulation and polysaccharide production by apigenin and tt-farnesol. J Antimicrob Chemother 2003;52:782–789. doi: 10.1093/jac/dkg449 14563892 10.1093/jac/dkg449

[30] Aires CP, Del Bel Cury AA, Tenuta LM, Klein MI, Koo H, Duarte S, et al Effect of starch and sucrose on dental biofilm formation and on root dentine demineralization. Caries Res. 2008;42(5):380–6. doi: 10.1159/000154783 18781066 10.1159/000154783

[31] Dubois M, Gilles KA, Hamilton JK, Rebers PA, Smith F. Colorimetric method for determination of sugar and related substances. Anal Chem 1956;28:350–356.

[32] Vogel GL, Chow LC, Brown WE. A microanalytical procedure for the determination of calcium, phosphate and fluoride in enamel biopsy samples. Caries Res 1983;17:23–31. 6571804 10.1159/000260645

[33] Carvalho-Netto OV, Carazzolle MF, Mofatto LS, Teixeira PJ, Noronha MF, Calderón LA, et al *Saccharomyces cerevisiae* transcriptional reprograming due to bacterial contamination during industrial scale bioethanol production. Microb Cell Fact 2015;30;14:13 doi: 10.1186/s12934-015-0196-6 25633848 10.1186/s12934-015-0196-6 PMC4318157

[34] Koo H, Xiao J, Klein MI, Jeon JG. Exopolysaccharides produced by Streptococcus mutans glucosyltransferases modulate the establishment of microcolonies within multispecies biofilms. J Bacteriol. 2010 6;192(12):3024–32. doi: 10.1128/JB.01649-09 20233920 10.1128/JB.01649-09 PMC2901689

[35] Xiao J, Koo H. Structural organization and dynamics of exopolysaccharide matrix and microcolonies formation by *Streptococcus mutans* in biofilms. J Appl Microbiol 2010;108:2103–2113. doi: 10.1111/j.1365-2672.2009.04616.x 19941630 10.1111/j.1365-2672.2009.04616.x

[36] Guggenheim M, Shapiro S, Gmür R, Guggenheim B. Spatial arrangements and associative behavior of species in an in vitro oral biofilm model. Appl Environ Microbiol. 2001 3;67(3):1343–50. doi: 10.1128/AEM.67.3.1343-1350.2001 11229930 10.1128/AEM.67.3.1343-1350.2001 PMC92733

[37] Lucena-Ferreira SC, Ricomini-Filho AP, Silva WJ, Cury JA, Cury AA. Influence of daily immersion in denture cleanser on multispecies biofilm. Clin Oral Investig. 2014;18(9):2179–2185. doi: 10.1007/s00784-014-1210-9 24590620 10.1007/s00784-014-1210-9

[38] Hartig SM. Basic image analysis and manipulation in ImageJ. Curr Protoc Mol Biol 2013;14:1–14. 10.1002/0471142727.mb1415s102 23547012

[39] Heydorn A, Nielsen AT, Hentzer M, Sternberg C, Givskov M, Ersbøll BK, et al Quantification of biofilm structures by the novel computer program COMSTAT. Microbiology. 2000 10;146 (Pt 10):2395–407. 11021916 10.1099/00221287-146-10-2395

[40] Vorregaard M. Comstat2—a modern 3D image analysis environment for biofilms, in Informatics and Mathematical Modelling. Technical University of Denmark: Kongens Lyngby, Denmark, 2008.

[41] Cury JA, Francisco SB, Del Bel Cury A, Tabchoury CPM. In situ study of sucrose exposure, mutans streptococci in dental plaque and dental caries. Braz Dent J 2001 12:101–104. 11445910

[42] Klein MI, Duarte S, Xiao J, Mitra S, Foster TH, Koo H. Structural and molecular basis of the role of starch and sucrose in *Streptococcus mutans* biofilm development. Appl Environ Microbiol. 2009;75:837–841. doi: 10.1128/AEM.01299-08 19028906 10.1128/AEM.01299-08 PMC2632160

[43] Xiao J, Klein MI, Falsetta ML, Lu B, Delahunty CM, Yates JR 3rd, et al The exopolysaccharide matrix modulates the interaction between 3D architecture and virulence of a mixed-species oral biofilm. PLoS Pathog 2012;8:e1002623 doi: 10.1371/journal.ppat.1002623 22496649 10.1371/journal.ppat.1002623 PMC3320608

[44] Dawes C. What is the critical pH and why does a tooth dissolve in acid? J Can Dent Assoc. 2003 12;69(11):722–4. 14653937

